# Effect of Benfotiamine in Podocyte Damage Induced by Peritoneal Dialysis Fluid

**DOI:** 10.3389/fmed.2015.00010

**Published:** 2015-03-10

**Authors:** Sandra Müller-Krebs, Katharina Nissle, Julia Tsobaneli, Martin Zeier, Lars Philipp Kihm, Zoltan Kender, Thomas Fleming, Peter Paul Nawroth, Jochen Reiser, Vedat Schwenger

**Affiliations:** ^1^Department of Nephrology, University of Heidelberg, Heidelberg, Germany; ^2^Department of Endocrinology, University of Heidelberg, Heidelberg, Germany; ^3^2nd Department of Medicine, Semmelweis University, Budapest, Hungary; ^4^Department of Medicine, Rush University Medical Center, Chicago, IL, USA

**Keywords:** benfotiamine, glucose degradation products, high glucose, peritoneal dialysis, peritoneal dialysis fluids, podocytes, systemic toxicity

## Abstract

**Background:** In peritoneal dialysis (PD), residual renal function (RRF) fundamentally contributes to improved quality of life and patient survival. High glucose and advanced glycation end-products (AGE) contribute locally to peritoneal and systemically to renal damage. Integrity of podocyte structure and function is of special importance to preserve RRF. Benfotiamine could counteract the glucose and AGE-mediated toxicity by blocking hyperglycemia-associated podocyte damage via the pentose-phosphate pathway.

**Methods:** A human differentiated podocyte cell line was incubated with control solution (control), 2.5% glucose solution (glucose), and 2.5% peritoneal dialysis fluid (PDF) for 48 h either ±50 μM benfotiamine. Podocyte damage and potential benefit of benfotiamine were analyzed using immunofluorescence, western blot analysis, and a functional migration assay. For quantitation, a semiquantitative score was used.

**Results:** When incubating podocytes with benfotiamine, glucose, and PDF-mediated damage was reduced, resulting in lower expression of AGE and intact podocin and ZO-1 localization. The reorganization of the actin cytoskeleton was restored in the presence of benfotiamine as functional podocyte motility reached control level. Decreased level of inflammation could be shown as well as reduced podocyte apoptosis.

**Conclusion:** These data suggest that benfotiamine protects podocytes from glucose and PDF-mediated dysfunction and damage, in particular, with regard to cytoskeletal reorganization, motility, inflammation, and podocyte survival.

**Short summary**: It is known that high glucose and advanced glycation end-products (AGE) do not only cause local peritoneal toxicity but also systemic damage and thereby cause a loss of residual renal function (RRF). When treating podocytes with the vitamin B1 analog benfotiamine, damage mediated by high glucose and peritoneal dialysis (PD) fluids is reduced, in particular, regarding cytoskeletal organization, motility, inflammation, as well as podocyte survival; this might be an implication for preservation of RRF in PD.

## Introduction

Preservation of residual renal function (RRF) contributes significantly to an improved survival of patients undergoing peritoneal dialysis (PD) (1–3). Several studies have tried to identify potential risk factors for the decline of RRF, but the underlying mechanisms remain unknown. Glucose and glucose degradation products (GDP), present at higher concentrations in the peritoneal dialysis fluid (PDF), have been shown to be a major mediator of damage to the peritoneum (4–6). As a consequence of reabsorption, the effects of the PDFs are not limited to the peritoneal cavity, and studies have shown that the kidneys are particularly susceptible, leading to damage, and decreased RRF (7–11).

Glucose degradation products are a heterogeneous group of small molecular weight carbonyls formed within PDFs as a direct consequence of heat-sterilization (12). They exhibit a facile reactivity with various biomolecules, including proteins, DNA, and phospholipids, generating stable products, such as advanced glycation end-products (AGEs). An accumulation of AGEs has been shown to contribute to the pathogenesis of vascular diseases, such as atherosclerosis and diabetes (13). Furthermore, AGE-modified proteins have also been shown to interact and activate the receptor for AGE (RAGE), leading to an NFκB dependent pro-inflammatory response. It could be demonstrated that blocking of AGE/RAGE interactions in podocytes, using an anti-human RAGE body, was able to prevent GDP-induced inflammation (14). Although effective *in vitro*, the use of blocking antibodies *in vivo* and particularly as a clinical treatment option is imperfect.

An alternative strategy would be to reduce the burden of the high glucose and GDP content in PDFs through activation of innate defensive metabolic pathways, such as the pentose-phosphate pathway. It has been demonstrated that the peritoneal membrane can be protected against PDF-induced damage by high glucose, by treatment with benfotiamine, an analogy of thiamine, and cofactor for transketolase (TKT). Increased TKT activity, *in vitro* and *in vivo*, has been shown to be effective in reducing the accumulation of the triosesphosphate intermediates (glyceraldeyhde-3-phosphate and dihydroxyacetone phosphate), by activation of the pentose-phosphate pathway, shifting glycolytic flux into the formation of ribose-5-phosphate and NADPH. Reducing the accumulation of the triosesphosphate pool, prevents the activation of the classical biochemical pathways responsible for cellular dysfunction observed in hyperglycemia, such as the increased *de novo* synthesis of diacylglycerol and activation of protein kinase C (PKC); oxidative stress linked to mitochondrial dysfunction; activation of the hexosamine pathway, and the increased formation of methylglyoxal (MG) and AGEs (9).

The purpose of the study was to investigate the effects of benfotiamine to glucose and PDF in preventing cellular dysfunction in podocytes.

## Materials and Methods

### Cell culture of podocytes

The conditionally immortalized human podocyte cell line AB8/13 was cultured as described elsewhere (15). In brief, podocytes were maintained in RPMI-1640 medium supplemented with 10% fetal calf serum (FCS; Gibco-BRL, Gaithersburg, MD, USA), 100 U/mL penicillin, 100 mg/mL streptomycin, 1% insulin–transferrin–selenium liquid media supplement (Sigma-Aldrich, Taufkirchen, Germany), and 10 IU/mL recombinant mouse interferon-γ (Cell Sciences, Canton, MA, USA) at 33°C (permissive conditions). To induce differentiation, podocytes were cultured at 37°C without interferon-γ (non-permissive conditions). After 14 days under non-permissive conditions, cells had an arborous shape and expressed synaptopodin and podocin, as determined by immunofluorescence or western blot analysis (*Data not shown*). Passages between 5 and 15 were used in this study.

### Incubation of podocytes with high glucose, PDF, and benfotiamine

Differentiated podocytes, grown on type I collagen coated plates, were starved in RPMI containing 0.5% FCS for 24 h. The cells were then treated with high glucose (glucose) or PDF for 48 h. High glucose concentration was 2.5%; as PDF, the 2.5% glucose containing Gambrosol Trio 10 (GambroCorporate Research, Hechingen, Germany) was used. All experiments were repeated at least three times in each indicated condition. Glucose and PDF were used for incubation in a ratio with respect to cell culture medium 1 + 1 (9, 14, 16, 17). For control stimulations, cells were treated with a buffer-only compartment of the PDF, contenting no glucose (kindly provided by Gambro, Lundia, Sweden). Cells were co-stimulated with or without benfotiamine (WörwagPharma, Böblingen, Germany), to final concentration of 50 μM as performed before (9, 13). To exclude different protein content due to different number of cells after incubation with glucose and PDF, we measured the protein content of cell lysates of all groups using PierceBCA Protein Assay Kit (Thermo Scientific, Langenselbold, Germany) according to the manufacturer’s instructions.

### Immunofluorescence

Following stimulation, cells were either fixed with methanol or 3% paraformaldehyde phosphate-buffered saline (PBS) for 15 min, permeabilized with 0.2% Triton X-100 for 5 min, and incubated with primary antibodies and appropriate fluorescently labeled secondary antibodies in blocking solution (2% bovine serum albumin, 2% FCS, and 0.2% fish gelatine in PBS). For nuclear staining and detection of fragmented apoptotic nuclei, fixated cells were stained with 0.1 mg/mL Hoechst 33342 (Invitrogen, Karlsruhe, Germany), which was included with the secondary antibody. Antibodies directed against the following proteins were used: anti-AGE (rabbit polyclonal IgG, BioLogo, Kronshagen, Germany), anti-synaptopodin (P-19) (goat polyclonal IgG), anti-podocin (H-130) (rabbit polyclonal IgG), anti-nuclear factor kappa B (NFκB) P65 (rabbit polyclonal IgG, Santa Cruz Biotechnology, Heidelberg, Germany), and anti-zonula occludens-1 (ZO-1) (rabbit polyclonal IgG, Invitrogen, Karlsruhe, Germany). With the exception of the anti-phalloidinIgG (Sigma-Aldrich, Deisenhofen, Germany), which was directly conjugated to fluorescein isothiocyanate (FITC), for antigen–antibody complexes were visualized with DyLight 488- and Cy3-conjugated secondary antibodies (Dianova, Hamburg, Germany). Negative control was performed by using PBS instead of primary antibody.

To investigate AGE expression, a semiquantitative analysis was carried out using a score system of 0–4 (0 = no signal to 4 = strong signal). For analysis of actin cytoskeleton organization, the number of cells, which revealed a cortical reorganization of the actin cytoskeleton, was counted, and the total cell number was set to 100%. For analysis of NFκB–p65 activation, a positive signal, which co-localized with the nucleus, was counted, and the total cell number set to 100%. Localization of podocin within the cell was analyzed using a scoring system that comprises membrane/cytoplasmic pattern (score 0), beginning reorganization to perinuclear or nuclear envelope (score = 1), and perinuclear or nuclear envelope (score = 2). Apoptosis was examined by Hoechst-stained fragmented nuclei or apoptotic bodies, percentage of apoptotic cells were assessed, control − benfotiamine was set zero and fold change shown.

Images were taken using a Nikon DS-Qi1Mc quantitative black-and-white charge-coupled device camera attached to a Nikon Eclipse 80i upright microscope (Nikon, Düsseldorf, Germany). The same contrast and intensity settings were applied to samples stained with identical antibodies (9, 14, 18, 19).

### Western blot analysis

Following stimulation, podocytes were lysed, protein concentration was assessed by Pierce BCA Protein Assay Kit (Thermo Scientific, Langenselbold, Germany) and after being boiled in sodium dodecyl sulfate (SDS) sample buffer, 5–20 μg of lysate was separated by 8–12% SDS-polyacrylamide gel electrophoresis and transferred to a nitrocellulose membrane. The following antibodies were used: anti-podocin (H-130) (rabbit polyclonal IgG, Santa Cruz Biotechnology, Heidelberg, Germany) and anti-ZO-1 (rabbit polyclonal IgG, Invitrogen, Karlsruhe, Germany). Equal loading was verified by the detection of tubulin with anti-α-tubulin (mAB IgG1, Sigma-Aldrich, Deisenhofen, Germany). Protein detection was performed after the incubation with primary and peroxidase-conjugated secondary antibodies using a Supersignal Pico detection kit (Pierce, Bonn, Germany) according to the manufacturer’s instructions.

### Migration assay

A *scratch*, using P200 tip was made within the monolayer of differentiated podocytes and treated as described. Images were taken immediately after wounding (0 h) and at 24 h after using a motor-controlled inverse microscope system Nikon Eclipse TE2000-E (Nikon, Düsseldorf, Germany). Analyses were performed with NIS Elements AR 2.30 (Nikon, Düsseldorf, Germany) by counting the number of cells that had migrated into the sized fields (14, 19, 20). For statistical analysis, the wounded area from each dish was measured in duplicates at six random wound gap locations per frame recorded per experiment, and at least four independent scratch-wound experiments were used for calculations.

### Statistical analysis

All data are demonstrated as mean ± SD of at least three independent experiments unless otherwise specified. The Mann–Whitney *U*-test was carried out to test statistical significance. The significance level was set at least at *P* < 0.05. The statistical analysis was performed by PC-Statistik (version 5.0; Hoffmann, Giessen, Germany) and Graph-Pad Prism (version 4.03; San Diego, CA, USA).

## Results

### AGE formation

**Figure 1:**
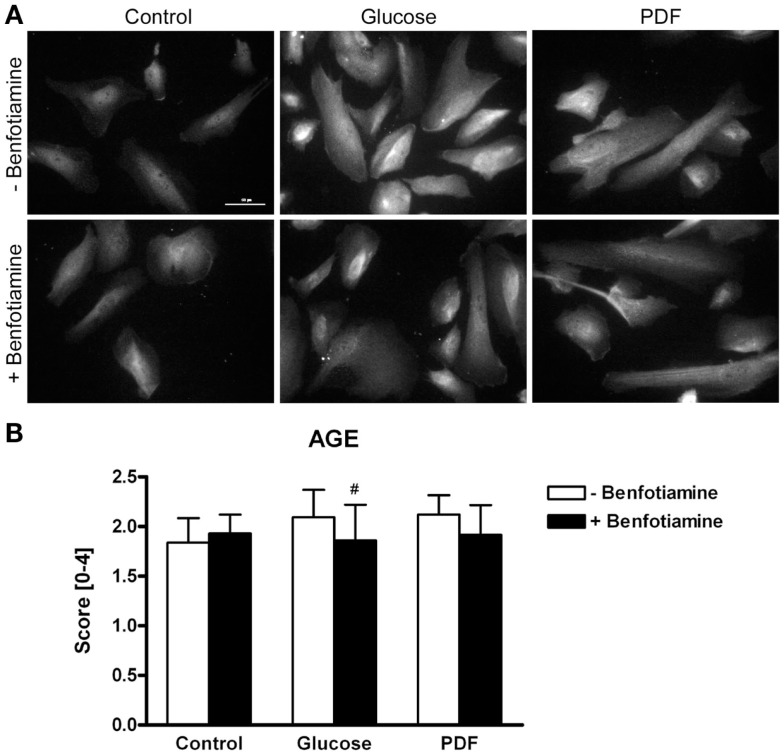
**AGE expression**. Immunofluorescence staining of podocytes to investigate the effect of ±benfotiamine after 48 h of incubation on the expression of AGE in the control, glucose, and PDF group. Highest AGE expression was found in the glucose and PDF group without benfotiamine; scale bar represents 50 μm **(A)**. Quantitation of the AGE expression, highest AGE expression was present in the glucose and PDF group without benfotiamine **(B)**. ^#^*P* < 0.05 versus −benfotiamine. AGE, advanced glycation end-products; PDF, peritoneal dialysis fluid.

Immunofluorescence analysis revealed a stronger total AGE formation in podocytes incubated with glucose or PDF. In contrast, addition of benfotiamine resulted in decreased AGE formation and deposition in podocytes (Figures 1A,B).

### Localization of podocyte specific protein podocin

**Figure 2:**
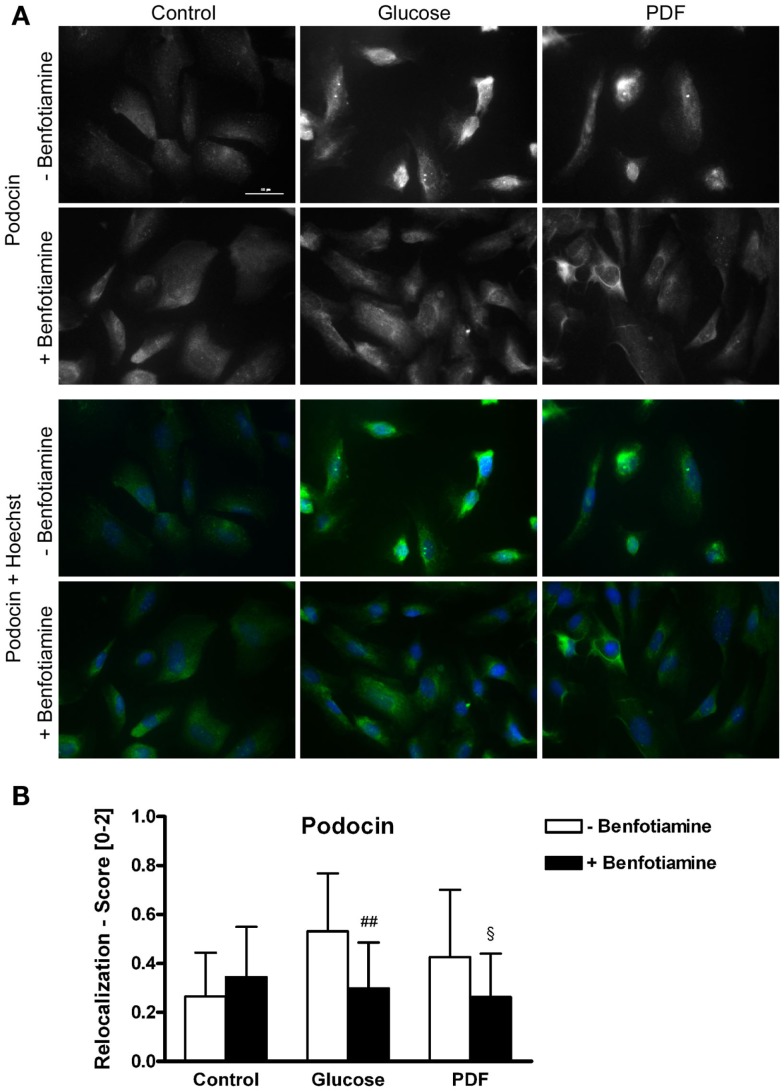
**Localization of podocin**. Immunofluorescence staining of podocytes to investigate the effect of ±benfotiamine after 48 h of incubation on the localization of podocin in the control, glucose, and PDF group. Highest relocalization score was observable in the glucose and PDF group without benfotiamine. Representative images of podocin staining (Dylight488), upper panel, and a merge of podocin and nuclei (Hoechst), lower panel, scale bar represents 50 μm **(A)**, quantitation of the relocalization of podocin **(B)**. ^##^*P* < 0.01 versus −benfotiamine, ^§^*P* < 0.05 versus −benfotiamine. PDF, peritoneal dialysis fluid.

It is known that mutations in the podocin gene NPHS2 cause nephrotic syndrome that is accompanied by proteinuria (21). Here, we found a relocalization of podocin protein after incubation of glucose and PDF without benfotiamine. These groups exhibited significantly more cells localized perinuclearly or to the nuclear envelope in comparison to when benfotiamine was added and as compared to control cells (score: glucose: 0.53 ± 0.24 versus glucose + benfotiamine: 0.30 ± 0.19, *P* < 0.01; PDF: 0.43 ± 0.27 versus PDF + benfotiamine: 0.26 ± 0.18, *P* < 0.05, Figures 2A,B).

### Localization of zonula occludens protein-1

**Figure 3:**
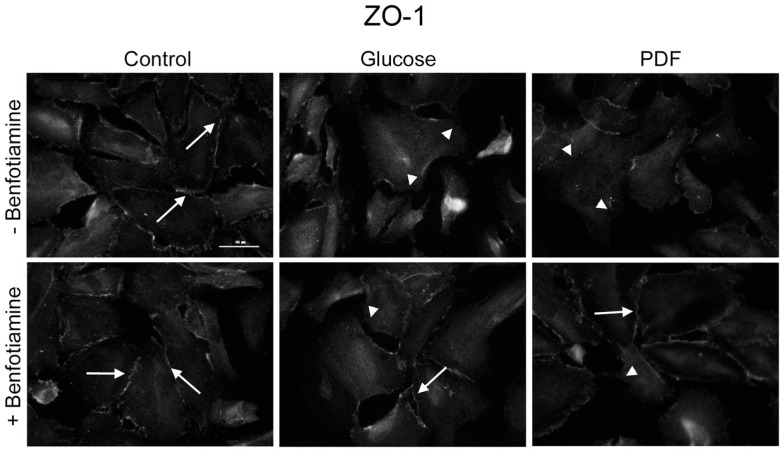
**Localization of zonula occludens protein-1**. Immunofluorescence staining of podocytes to investigate the effect of ±benfotiamine after 48 h of incubation on the localization of ZO-1 in the control, glucose, and PDF group. Highest relocalization was observable in the glucose and PDF group without benfotiamine. Representative images of ZO-1 staining, scale bar represents 50 μm; arrows show normal localization of ZO-1; arrow heads show mislocalized/absent of ZO-1. ZO-1, zonula occulens-1; PDF, peritoneal dialysis fluid; C, control group; C + B, control group + benfotiamine; G, glucose group; G + B, glucose group + benfotiamine; P, PDF group; P + B, PDF group + benfotiamine.

To investigate localization of tight junction-associated protein ZO-1 an immunofluorescence analysis was performed. It is known that in differentiated podocytes ZO-1 localizes to sites of cell–cell contacts between interdigitating processes (22). There was decreased ZO-1 localization at sites of cell–cell contact in the glucose and PDF group; in the presence of benfotiamine, a normal distribution of ZO-1 at the cell–cell contacts was maintained (Figure 3).

### Actin cytoskeleton

**Figure 4:**
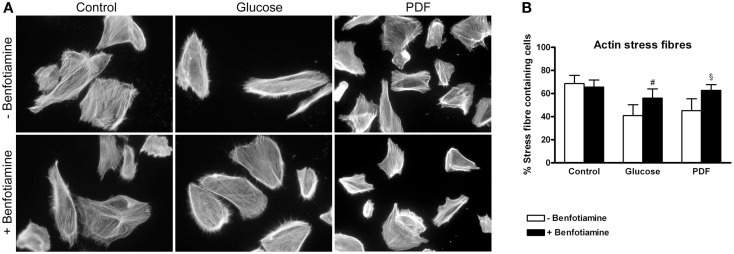
**Actin cytoskeleton**. Immunofluorescence staining of podocytes to investigate the effect of ±benfotiamine after 48 h of incubation on phalloidin in the control, glucose, and PDF group, magnification 400 x **(A)**. Quantitation of the organization of the actin cytoskeleton, lowest percentage of normal stress fiber organization was present in the glucose and PDF group without benfotiamine **(B)**. ^#^*P* < 0.05 versus −benfotiamine, ^§^*P* < 0.05 versus −benfotiamine. PDF, peritoneal dialysis fluid.

The preservation of the actin cytoskeleton is important to maintain morphology and function of podocytes. Actin fiber status can be analyzed with respect to normal well-developed podocyte stress fibers or a reorganized cortical actin fiber phenotype (14, 23). For this, immunofluorescence staining of phalloidin was conducted. In our experiments, the control group (±benfotiamine) showed well-developed stress fibers whereas a distinct increase in rearranged fibers could be found in the glucose and PDF group without benfotiamine. Here, we observed a cortical actin fiber phenotype. In contrast, addition of benfotiamine resulted in a significant increase of normal stress fibers, although not reaching control level (glucose: 41.0 ± 9.31% versus glucose + benfotiamine: 56.0 ± 8.04%, *P* < 0.05; PDF: 45.2 ± 10.3% versus PDF + benfotiamine: 62.69 ± 5.00%, *P* < 0.05, Figures 4A,B).

### Migration assay

**Figure 5:**
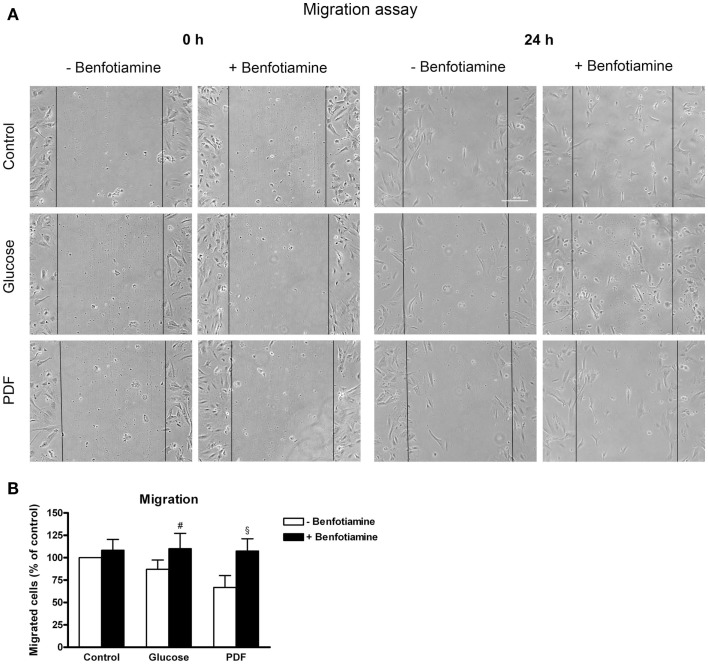
**Migration**. Migration assay to investigate the effect of ±benfotiamine after 48 h of incubation on podocyte motility in the control, glucose, and PDF group, phase contrast microscopy, scale bar represents 200 μm **(A)**. Quantitation of migration, lowest motility was found in the glucose and PDF group without benfotiamine **(B)**. ^#^*P* < 0.05 versus −benfotiamine, ^§^*P* < 0.05 versus −benfotiamine. PDF, peritoneal dialysis fluid.

The wound was closed best in the control groups (±benfotiamine). In contrast, in the glucose and PDF (−benfotiamine) migration was significantly reduced, whereas when benfotiamine was present, motility was augmented to control level (migrated cells – % of control: glucose: 87.1 ± 10.3% versus glucose + benfotiamine: 110 ± 17.2%, *P* < 0.05; PDF: 66.8 ± 13.2% versus PDF + benfotiamine: 107 ± 13.8%, *P* < 0.05, Figures 5A,B).

### Inflammation – NFκB activation

**Figure 6:**
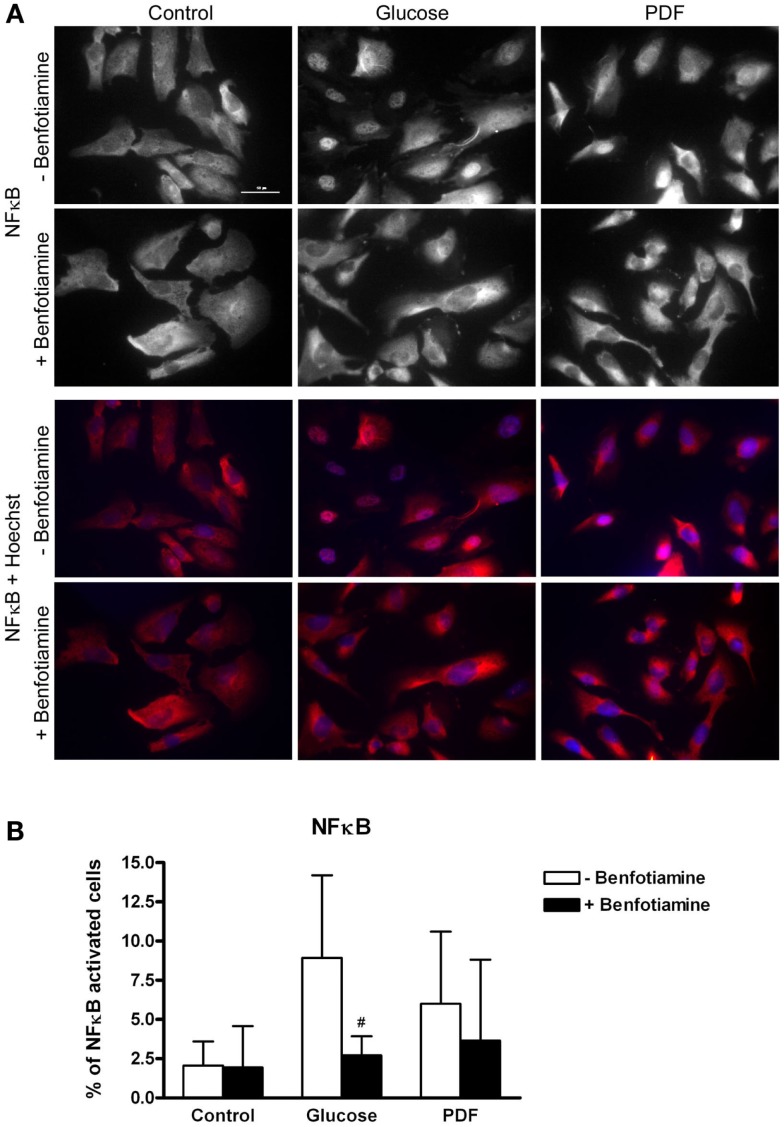
**Inflammation – NFκB activation**. Immunofluorescence staining of podocytes to investigate the effect of ±benfotiamine after 48 h of incubation on inflammation in the control, glucose, and PDF group, representative images of NFκB–P65 staining, (Cy3) upper panel, and a merge of NFκB and nuclei (Hoechst), lower panel, scale bar represents 50 μm **(A)**. Quantitation of NFκB activated cells, highest percentage of NFκB activation was present in the glucose and PDF group without benfotiamine **(B)**. ^#^*P* < 0.05 versus −benfotiamine. NFκB, nuclear factor kappa B; PDF, peritoneal dialysis fluid.

Higher numbers of NFκB activated cells shown by nuclear NFκB expression could be observed in the glucose and PDF group compared to control. This NFκB activation was reduced when benfotiamine had been added (glucose: 8.93 ± 5.26% versus glucose + benfotiamine: 2.72 ± 1.21%, *P* < 0.05; PDF: 6.01 ± 4.60% versus PDF + benfotiamine: 3.66 ± 5.17%, Figures 6A,B).

### Apoptosis

**Figure 7:**
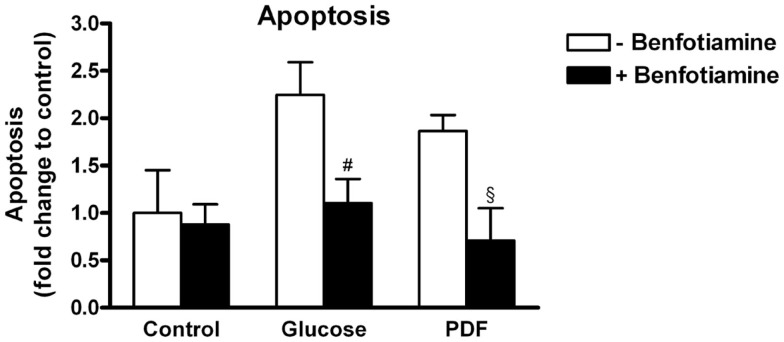
**Apoptosis**. Analysis of apoptosis in podocytes to investigate the effect of ±benfotiamine after 48 h of incubation in the control, glucose, and PDF group. Apoptosis was detected by fragmented nuclei and was highest in the glucose and PDF group. *^#^P* < 0.05 versus −benfotiamine, ^§^*P* < 0.05 versus −benfotiamine.

This morphological analysis revealed about twofold higher apoptosis in the glucose group and the PDF group in comparison to control. When benfotiamine had been added apoptosis rates were significantly reduced in both groups (fold control: glucose: 2.25 ± 0.34 versus glucose + benfotiamine: 1.10 ± 0.26, *P* < 0.05; PDF: 1.86 ± 0.17 versus PDF + benfotiamine: 0.71 ± 0.34%, *P* < 0.05, Figure 7).

## Discussion

During PD, exposure to high glucose and GDP-containing PDF are the causative agents leading to peritoneal and systemic toxicity (4, 5, 8, 24). Although clinical studies failed to identify high GDP-containing PDF as a valid risk factor contributable for the decline of RRF in PD (25), animal studies support the hypothesis that highly reactive GDP might be major mediators of renal damage (8).

Within this context alternative therapeutic approaches to reduce the burden of high glucose and GDP content in PDF are of special interest. Many approaches have been taken to lower the toxicity of PDF, such as blocking TGFβ1 or the administration of anti-fibrous drugs, such as rosiglitazone. Benfotiamine has also been shown to be beneficial in this regard (9). Benfotiamine is a thiamine monophosphate prodrug, which activates the pentose-phosphate pathway by increasing the activity of TKT (13, 26). It has been shown that expression of TKT is a critical factor in preventing hyperglycemia-induced metabolic dysfunction *in vitro* (27). Activation of pentose-phosphate pathway not only leads to NADPH production, which helps to sustain enzymatic antioxidant defenses as well the metabolism of reactive metabolites formed from oxidative and non-oxidative processes, but also prevents the accumulation of fructose-6-phosphates and triosesphosphates, thereby preventing the activation of the hexamine and PKC pathways, as well as mitochondrial dysfunction driven by the glycerophosphate shuttle and increased formation of MG and MG-associated advanced glycation end-products (AGEs) (13, 28).

As benfotiamine interferes into the glucose metabolism at an early stage, by shifting glycolytic flux away from the accumulation of triophosphates into the formation of ribose-5-phosphate (13, 26), it could potentially reduce podocyte toxicity, particularly as it has been shown that glucose and PDF enter the systemic circulation and thereby cause podocyte damage (7, 8, 14). The purpose of our study was to investigate the potential beneficial effects of benfotiamine to podocyte dysfunction during PD; therefore, we cultured human podocytes and incubated them with glucose and PDF in the absence and presence of benfotiamine. It was shown that treatment with high glucose and/or GDP-containing PDF increased AGE formation, as well relocalization of proteins such as podocin protein to the perinucleus and/or nuclear envelope; decreased ZO-1 from sites of cell–cell contact and reorganization of actin cytoskeleton to a cortical fiber phenotype. These changes were associated with decreased cell migration, increased activation of NFκB, and increased apoptosis. These observed effects were reduced by co-treatment with benfotiamine.

Although no direct measurements of TKT activity or the pentose-phosphate pathway were made, the observed reduction in AGE formation and deposition would tend to suggest that the beneficial effects observed are likely due to the activation of the pentose-phosphate pathway. Further studies would be required to confirm this. These findings also would suggest within the context of PD that use of benfotiamine would be an effective therapeutic option for reducing the toxic effects of PDF (9) and preserving RRF; however, its usefulness would be limited to only addressing the direct effects of high glucose from PDF and not the effects of the high toxic GDPs, which are also present at high concentrations.

Studies have shown that the toxic effects of GDPs can be reduced by supplementing PDFs with either scavengers of GDPs, such as aminoguanidine, or antioxidants, such as glutathione. Furthermore, it has also been shown that blocking of the RAGE can prevent GDP-induced cellular damage in podocytes incubated with PDF (14). Therefore, any new therapeutic treatments to preserve RRF in PD patients would need to target not only the effects of high glucose but also the effects of GDPs. This is supported by clinical studies, which have failed to show a clear association between declining RRF in PD and glucose or GDP content of the PDFs.

Further *in vitro* and *in vivo* studies would be required to establish whether a treatment with benfotiamine or in combination with either antioxidants and/or scavengers of GDPs would be an effective approach to reducing the toxic effects of PDF. Especially in clinical studies, we plan to proof our hypothesis, as benfotiamine is a vitamin B1 analog that might be an applicable drug to be administered to patients with negligible side effects to prevent local and systemic damage in PD.

## Conclusion

The findings of this study suggest that treatment with benfotiamine can reduce the toxic effects of PDFs, in particularly those affects resulting from high glucose. Further experimental and clinical studies are required to validate whether treatment with benfotiamine would be of therapeutic benefit to patients undergoing PD.

## Conflict of Interest Statement

We have had no involvements that might raise the question of bias in the work reported or in the conclusions, implications, or opinions stated. The results presented in this paper have not been published previously in whole or in part, except in abstract form. Vedat Schwenger and Sandra Müller-Krebs received a research grant from WörwagPharma (Böblingen, Germany).
